# A Mobile Multi-Agent Information System for Ubiquitous Fetal Monitoring

**DOI:** 10.3390/ijerph110100600

**Published:** 2014-01-02

**Authors:** Chuan-Jun Su, Ta-Wei Chu

**Affiliations:** 1 Department of Industrial Engineering & Management, Yuan Ze University, No. 135, Yuan-Tung Rd., Chung-Li City, Taoyuan County 320, Taiwan; 2 Department of Obstetrics and Gynecology, Tri-service General Hospital, National Defense Medical Center (Taipei MJ Health Screening Center), Taipei 100, Taiwan; E-Mail: taweichu@gmail.com

**Keywords:** mobile agent technology, Java Agent Development Environment (JADE), Electronic Fetal Monitoring (EFM), automated diagnosis

## Abstract

Electronic fetal monitoring (EFM) systems integrate many previously separate clinical activities related to fetal monitoring. Promoting the use of ubiquitous fetal monitoring services with real time status assessments requires a robust information platform equipped with an automatic diagnosis engine. This paper presents the design and development of a mobile multi-agent platform-based open information systems (IMAIS) with an automated diagnosis engine to support intensive and distributed ubiquitous fetal monitoring. The automatic diagnosis engine that we developed is capable of analyzing data in both traditional paper-based and digital formats. Issues related to interoperability, scalability, and openness in heterogeneous e-health environments are addressed through the adoption of a FIPA2000 standard compliant agent development platform—the Java Agent Development Environment (JADE). Integrating the IMAIS with light-weight, portable fetal monitor devices allows for continuous long-term monitoring without interfering with a patient’s everyday activities and without restricting her mobility. The system architecture can be also applied to vast monitoring scenarios such as elder care and vital sign monitoring.

## 1. Introduction

Rapid advances in Internet technologies in recent years have helped drive the growth of electronic fetal monitoring (EFM) systems. EFM systems leverage electronic apparatus and information technologies (IT) to support the collection and analysis of a variety of fetal monitoring information, along with related technologies and/or services. Before EFM systems were introduced in the 1950s, doctors and nurses could only periodically monitor the baby’s heartbeat using a stethoscope on the gravida’s abdomen [1]. While this approach helped obstetricians monitor fetal safety, it did not allow them to detect subtle changes in fetal heartbeats or offer continuous surveillance, thus obstetricians still lacked vital information for reducing perinatal or neonatal mortality rates.

The importance of fetal monitoring during labor is well recognized. Fetal monitors provide satisfactory and accurate monitoring of the heartbeat of the fetus and contractions of the uterus. The pattern of the fetus’s heart beat usually reflects its health [2]. The collected pattern data may be used to determine the status of the fetus during the prenatal stage or during the birth process. The fetal heart monitoring process can be performed ubiquitously following an agreed procedure [3,4,5]. After recording, the trace is transmitted via modem to the operations center over a public telephone line. Despite the benefits of fetal monitoring, the obstetrician usually needs to a keep constant watch over the output of fetal heart monitors and respond appropriately. In some cases the obstetrician may fail to notice a key change in data leading to misdiagnosis. Moreover, home-based fetal monitoring not only allows parents to access information about the development of their babies but also helps reduce the number of hospital trips required. Developing a distributed automated diagnostic mechanism can aid obstetricians and promote home-based fetal monitoring. Over the past few decades, electronic fetal monitoring systems have emerged as a very promising tool for use by midwives, obstetricians, and labor and delivery nursing staff. These systems have encouraged the integration of many clinical activities related to fetal monitoring but advanced technologies have not yet enabled the obstetrician to provide real time status examinations and information exchange, and a solution is required to provide wide-area e-monitoring for clients at remote locations through low bandwidth, high latency asynchronous transactions, and assuming unstable connection communication environments in which servers are highly distributed and heterogeneous. 

This paper focuses on the design and development of a mobile multi-agent based distributed information system to allow automatic fetal monitoring in real time from any location using a PDA, smart phone, laptop, or desktop computer. 

The proposed system analyzes fetal monitoring data recorded remotely according to the doctor’s guidelines while the gravida is at home or work, or otherwise engaged in daily activities. Mobile agent (MA) technology offers the possibility of automatically executing monitoring tasks with minimal human intervention. This allows obstetricians to concentrate their attention on other activities and save valuable medical resources because the Mobile Agent (MA) technology enables medical personnel to capture large volumes of fetal information that can immediately be analyzed and used as the basis for effective and efficient treatment decisions without requiring the physician and patient to occupy the same space.

This study makes two main contributions: first, we compiled standard qualitative guidelines into physician-customizable and machine-processable quantitative criteria to enable automatic ubiquitous fetal monitoring. Second, we developed a highly distributed IMAIS system based on an open, high performance, FIPA standard-compliant agent development platform (the Java Agent Development Environment or JADE), which can automatically inform obstetricians of abnormalities, promote pervasive fetal monitoring, and help conserve and extend medical resources. The system allows for immediate communication and exchange of real time data, thus improving fetus safety while relieving medical staff from tedious data monitoring tasks and reducing the need for repeated hospital visits by the gravidas. 

## 2. Literature Review

### 2.1. Background on Fetal Monitoring

Hammacher and Hewlett Packard began to produce the first commercially available electronic fetal monitor (EFM) in 1978 [6]. The apparatus was used to monitor the fetal heartbeat (or heart rate) and maternal uterine contractions (UC) during labor and mainly focused on showing the output of each heart cycle to allow physicians to differentiate between the first and second heart sounds. During labor, continuous monitoring of the fetal heart rate is useful for detecting fetal distress, and a precipitous drop in the heart rate after a contraction could imply imminent fetal death if delivery is not affected immediately.

EFM devices help prevent dystocia during labor, so they are often used for routine monitoring of women in labor. Beat-to-beat variability is described quantitatively by indices originating from invasive fetal electrocardiography which provides the fetal heart rate (FHR) signal in the form of a time event series. Thus fetal wellbeing can be significantly predicted by analyzing the FHR variability. Janusz *et al*. presented a method for FHR signal processing that ensures accurate extraction of the time series of events from the sampled signal by reducing FHR signal distortions related to duplicated and invalid samples [7]. Another advance made by [8] was the development of an artificial neural network based on the logical interpretation of fuzzy if-then rules for evaluating the risk of low fetal birth weight using the quantitative description of cardiotocography signals. Additional innovations in FHR detection has accelerated the advancement of EFM technology [9,10]. With the development of wireless communications technologies, portable monitoring devices can be used in the office, the clinic, and even in the home with fetal data transmitted from remote locations via telephone or modem for analysis of fetal wellbeing. 

### 2.2. Agents and Multi-Agent Systems

An agent is a software entity that continuously and autonomously performs user-assigned tasks within a particular restricted environment. A software agent’s capacity for autonomy is what distinguishes it from a general software program. The term “Mobile Agent” (MA) was popularized by the Telescript Language [11], developed by General Magic in 1994, and has since been widely recognized as a novel abstraction for structuring distributed communication applications. The benefits of MA consist of overcoming the limitations of client devices, customizability, higher survivability, asynchronous and autonomous computing, and local data access and interoperability:

(1) *Overcoming client device limitations.* Issues related to communication delays and throughput, memory size, processing power, and limited storage are more easily managed if the agent is located closer to the data source. For example, placing an agent closer to a database that has to be searched through a custom algorithm can improve performance over accessing data remotely. This is a classical argument for locality of reference. In addition, the local storage may be insufficient to temporarily store large amounts of data, or may suffer from inadequate network bandwidth or processing power.
(2) *Customizability.* The traditional client-server model has more trouble adapting to frequent changes, but MAs can easily be customized to meet specific user needs, and sent to the server where customized requests are executed. In this study, requests are sent by using SQL to the targeted MA server. The MA server represents a processor that accepts and executes accepted agents.
(3) *Higher survivability.* Because agents transfer code and are state-encapsulated within the MA abstraction, they have a higher degree of survivability compared to the client-server model.
(4) *Asynchronous and autonomous computing.* Mobility is increasingly important in terms of user requirements. Jobs started from mobile devices frequently need to continue while a user is disconnected. While it is possible to delegate this responsibility to a stationary proxy residing on a network, it is even more convenient to delegate it to an MA that will pursue its owner’s tasks even while the user is disconnected. The agent can be pulled back from its current location when the user is back online. MA are able to continuously interact with their execution environment asynchronously and autonomously in pursuit of their own goals.
(5) *Local data access and interoperability.* MA access the data locally, which presents at least two advantages: (1) reduced network traffic and (2) a better use of network resources. The migration of an MA within the network follows protocols concerning agent transport, interaction and security to ensure interoperability among MAs which might use different operating systems.

The emergence of MA technology is evidenced by, among other things, the large number of systems contained in the MA lists [12], which are maintained at the University of Stuttgart, Germany and provide an approximate census of MA systems currently available. MAs can be considered as an emerging design paradigm in distributed environments [13,14] to reduce bandwidth consumption for better network connectivity. 

Picco’s overview of the fundamental characteristics regarding migration are taken as the core support for mobility to ensure this new paradigm can deal with distributed problems, where component locations are dynamic [13]. Several benefits were explored to encourage the adoption of the MA, such as processing decentralization, and communication and computational resource optimization. The most prominent feature is the support for designing applications that interact with human users [15].

### 2.3. Multi-Agent System Applied to Health Care

In health care field, agent technology has been widely applied to improve the performance of information systems in terms of interoperability, scalability and reconfigurability [16]. De Meo *et al*. presented an HL7-aware multi-agent system that supports personalized patient access to health care services. The proposed system combines submitted queries with the corresponding patient profiles to identify services likely to satisfy patient needs and desires [17]. Wu *et al*. proposed a multi-agent web service framework based on a service-oriented architecture to support qualified and optimized medical data translation in an e-healthcare information system [18]. Vaidehi *et al*. presented a design for a health care monitoring system based on multi-agent and wireless sensor networks which can collect, retrieve, store and analyze patient vital signs. The multi-agent system is applied to manage these sensors and to collect and store data in a database [19].

Camarinha-Matos and Vieira presented an MA-based architecture for health care centers to remotely observe and help elderly people living alone at home [20]. Su and Wu designed a highly distributed information infrastructure—MADIP—by using the multi-agent and MA paradigm, which can automatically notify the responsible care-provider of abnormalities, offer remote medical advice, and perform continuous health monitoring as needed [21]. Kim proposed a methodology for the design and implementation of u-healthcare, linking distributed mobile agents with medical entities into a collaborative environment [22]. Recent research has discussed the benefits of using agent technology and applications in the health care domain [16]. Table 1 presents a comparison with similar studies highlight the differences/contributions of the proposed IMAIS. 

**Table 1:** Comparison of IMAIS and other proposed systems.

Works	Service Domain	Multi-Agent System	Mobile Agent Adopted
De Meo *et al*., 2011 [17]	Personalized e-Health service access	Yes	No
Wu *et al*., 2012 [18]	Medical data transmission	Yes	No
Vaidehi *et al.*, 2013 [19]	In-home monitoring	Yes	No
Camarinha-Matos and Vieira, 1999 [20]	In-home monitoring	Yes	Yes
Su and Wu, 2011 [21]	Health care monitoring	Yes	Yes
Kim, *in press* [22]	Ubiquitous health care systems	Yes	Yes
IMAIS	Ubiquitous fetal monitoring	Yes	Yes

## 3. Requirement Analysis and System Design

### 3.1. Requirement Analysis

The traditional fetal monitoring process is extremely time consuming and requires the gravidas to frequently travel to hospitals or obstetrics clinics, causing them considerable inconvenience. However, fetal distress detection usually requires frequent observation of vital signs (e.g., fetal heart rate [FHR], UC, electrocardiograms [ECG], and oxygen saturation by pulse oximetry [SpO2]). Given limited medical resources, health care providers face profound challenges in providing high quality EFM services to an expanding population. 

Moreover, current practice keeps scarce medical staff occupied observing physiology parameters on in a monitoring center on a 24/7 basis. It is difficult for obstetricians or midwives to simultaneously audit and interpret the massive amounts of fetal information while performing diagnostics, verifying resuscitation and conducting other aggressive medical interventions. Such distractions have led to complications, yet there is no way to release hospital personnel from this routine and time-consuming task despite already being severely understaffed and overburdened. Hospital staff must assess time-critical situations and make vital decisions. Thus there is an urgent need to develop a system that is capable of performing ubiquitous EFM automatically and autonomously for users who are usually mobile and in environments potentially characterized by low bandwidth, high latency, asynchronous transactions, and unstable connections.

Minimum system requirements are as follows:

(1) *Openness*: Each instance of IMAIS installation is situated at home with for use by a technophobic client typically using a non-standard monitoring device. The IMAIS thus must be easily deployable, configurable, and updatable. An open architecture is required, allowing for the integration of new devices and knowledge-based modules.
(2) *Modularity*: Due to the complexity of the medical monitoring domain, distributed and encapsulated expertise is critical to IMAIS viability. “Modularity” is thus another crucial requirement for IMAIS to achieve extensibility by partitioning functions into smaller logical units that can be modified, enhanced, or added.

To satisfy these requirements, this research proposes a mobile multi-agent information platform (IMAIS) in which MAs collect distributed fetal vital sign data, and to spontaneously inform the physician about abnormal situations in real time.

**Figure 1:**
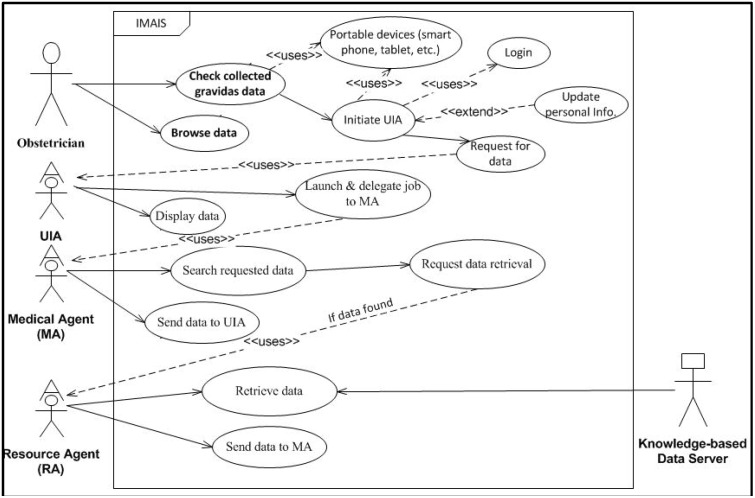
IMAIS use case diagram – obstetrician’s perspective.

**Figure 2:**
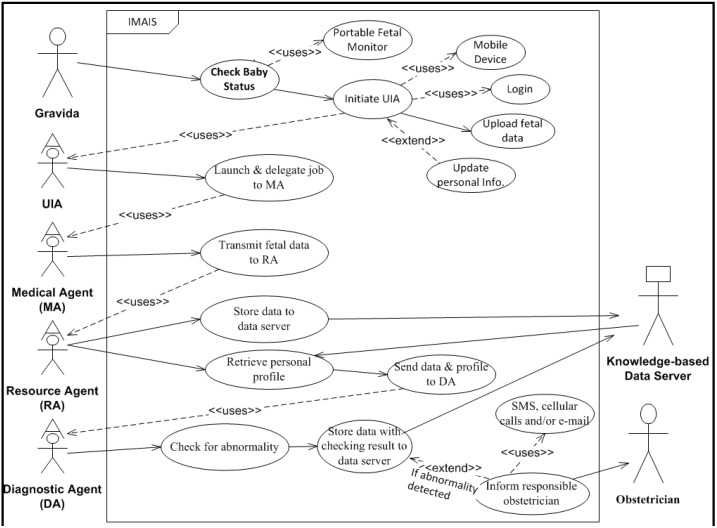
IMAIS use case diagram – gravida’s perspective.

To illustrate the user requirements, system functions and services, and system interaction with various user types, we developed two UML (Unified Modeling Language) case diagrams (Figure 1 and Figure 2). The use case diagrams serve as blueprints for IMAIS development and provide a simplified and graphical representation of a user’s interaction with the system.

### 3.2. System Design

EFM is a complex task that involves the sharing of technical knowledge, medical data, expertise, and services among women in labor and medical personnel. EFM integrates multiple systems with heterogeneous components, storing biochemical signals acquired from fetuses, and distributing physiologic information and resources. Given the intensive and distributed nature of the EFM environment, there is an urgent need to integrate disparate, stand-alone subsystems and their corresponding information repositories.

In this context, IMAIS is presented as a mechanism to support automatic and autonomous EFM. MAs can migrate from one system to another, and then operate on the target machine [23]. The migration characteristic accelerates the integration of isolated heterogeneous components. In addition, MAs are autonomous in that agents can control their own actions based on pre-established guidelines.

**Figure 3:**
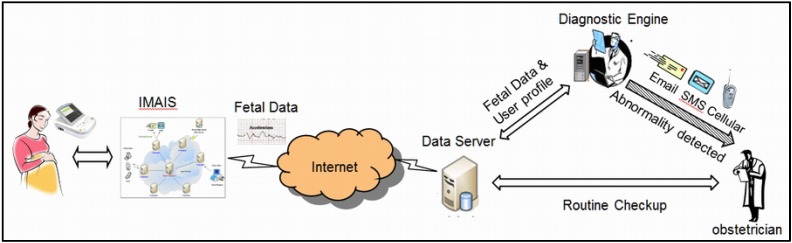
High level view of the preliminary IMAIS system design.

In the requirement elicitation phase, we solicited functional needs from interviews with various stakeholders including gravidas, nurses, and obstetricians. Their insights were incorporated in the system design phase. The interviews were organized by the author Dr. Ta-wei Chu, an experienced obstetrician. A high level view of the preliminary system design was subsequently developed by abstracting the complexity away from the end users as illustrated in Figure 3.

JADE was selected as our multi-agent systems framework for implementing IMAIS due to its Foundation for Intelligent Physical Agents (FIPA)-compliant interoperability, simplified development of distributed applications, openness, and its vast API library for the effective development of distributed mobile applications.

#### IMAIS Agent Environment

The Java Agent Development Framework (JADE) [24] agent platform is compliant with international Foundation for Intelligent Physical Agents (FIPA) [25] standards [26,27], and is thus used here as the foundation for building the IMAIS platform. In other words, the IMAIS agent environment, communication framework, and architecture are designed following the JADE specification.

An agent environment is composed of multiple containers which serve as the environment in which stationary agents reside and where mobile agents are created. In general, stationary agents stay in a specific execution environment, *i.e.*, an agent container, to provide mobile agents with resources and functionality [28]. While stationary agents remain static in an agent container, mobile agents migrate among agent networks to achieve their goals by taking advantage of resources in the local agent container.

**Figure 4:**
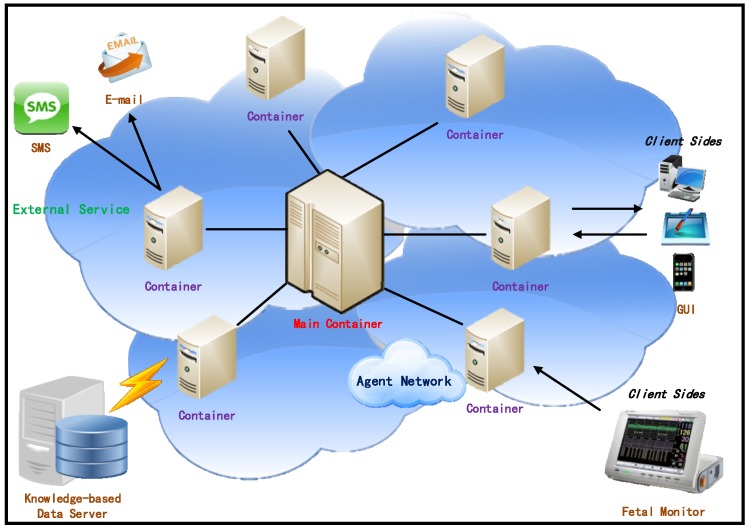
IMAIS agent environment.

The IMAIS agent environment consists of hosts running the agent containers and hosts supporting the knowledge-based data server and external services. As illustrated in Figure 4, agent containers can be dynamically added to and removed from an agent environment, thus allowing for large-scale, even network-wide, installations.

The main container is an essential component which manages all of the containers and agents in IMAIS. The other containers (“non-main containers”) are designed for various purposes such as the provision of external services (e.g., E-mail, SMS, *etc*.), and to establish the connection with the knowledge-based data server or the clients. Through external services (referred to as service-containers), agents may communicate with systems outside the agent environment. By working with the Knowledge-based Data Server, data containers are used to manage data provision and access. Client containers act as intermediaries that perform communication between users and the applications.

### 3.3. IMAIS System Architecture

**Figure 5:**
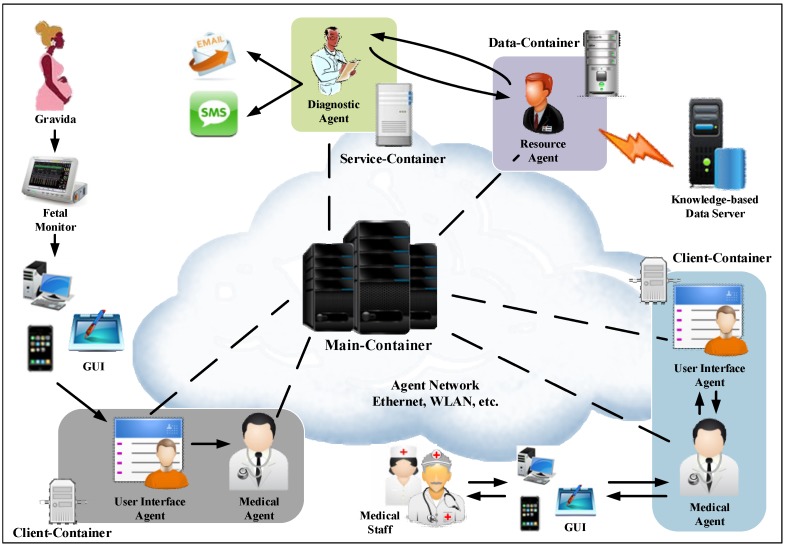
The infrastructure of an MA-based fetal monitoring platform.

The IMAIS system architecture consists of six primary architectural features: (1) User Interface Agent (UIA); (2) Medical (obstetrician) Agent; (3) Resource Agent; (4) Diagnostic Agent; (5) Knowledge-based Data Server; and (6) External Services. The infrastructure and the knowledge communication mechanism that enables agent collaboration are depicted in Figure 5. The explicit function of each agent is described below.

#### 3.3.1. User Interface Agent (UIA)

The UIA is a type of stationary agent that serves as the user’s gateway to the network of IMAIS agents. It is mainly responsible for routing user requests to the appropriate agents and returning feedback to the user. The UIA is persistent and autonomous, and can thus maintain the user’s context beyond a single browser session, allowing long-term autonomous data retrieval and other tasks to continue in the user’s absence. It receives the requests from the UI/application to invoke internal services. Hence, the UIA is an access control mechanism which authenticates users before starting a client-container. The UIA is responsible for the final presentation of results by a mobile agent or by a Diagnostic Agent before passing data to the application layer.

#### 3.3.2. Medical (Obstetrician) Agent

The Medical Agent is the MA used by the medical staff (e.g., obstetricians), enabling them to monitor labor conditions in real time. In this unit, medical staff members can use their mobile devices (e.g., GUI in Figure 5) to trigger Medical Agents as their delegates to perform monitoring tasks while the staff are engaged with other work. For a Medical Agent to be activated, it must be dispatched to a specific data-container. 

The agent network authenticates the incoming Medical Agent using its credentials and determines its privileges. It then assigns a thread to execute the Medical Agent which is then sent to the Internet through an access point (AP) to connect with an established wireless local area network (WLAN) in the hospital. After the agent is connected to the Internet, it will continue to perform its assigned monitoring task as needed according to the characteristics of the specific clinical case. The Medical Agent roams the networks to collect related fetal physiologic data, which it then carries back to its original client side host.

#### 3.3.3. Resource Agent

The Resource Agent is another type of stationary agent which normally operates at a higher level of trust and mediates access to resources from the MA to host computers. Resource Agents are “static” and do not migrate; they usually reside on the host computers to locally provide expert advice or services. Therefore, they are considered to be secure and granted access to resources on the host computer. In the proposed platform, the Resource Agent plays an important role in dynamically interfacing with the host’s resources. It acts as a front-end interface for the database, accepting high-level ACL-based queries from the network and translating them into local query language (e.g., SQL) for execution against the local database. Results are then translated back into the terms of the appropriate ontology and returned to the requesting agent. The mobile agent accesses host system resources through the Resource Agent, since mobile agents are restricted to communicating only with data containers and other agents. The Resource Agent takes the place of web sites to provide a bridge between users and the databases.

#### 3.3.4. Diagnostic Agent

The Diagnostic Agent analyzes data collected from fetal monitors. It first checks the collected data against criteria for fetal abnormality. If anomalies are detected, the Diagnostic Agent accesses external services, such as a short message service (SMS) or cellular phone to inform the associated obstetrician designated in the patient’s personal profile. The operations of the Diagnostic Agent are discussed in detail in Section 4.

#### 3.3.5. Knowledge-based Data Server

**Figure 6:**
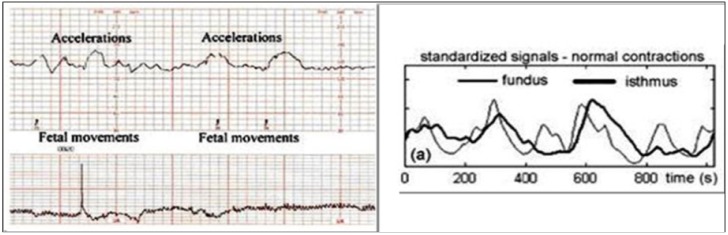
Fetal Heart Rate (FHR) and uterine contraction data stored in the data server.

It is evident that each fetus (or gravida) is an individual case with particular patterns, complications, and diseases. To optimize the performance of the proposed platform, it incorporates a Knowledge-based Data Server which consists of two information repositories: (1) “data” and (2) “user profiles”. The Medical Agent collects and transmits fetal monitoring data as shown in Figure 6 from a user to the Resource Agent that subsequently stores the data in the repository. A copy of the fetal monitoring data along with the user’s profile is also sent to the Diagnostic Agent for real-time monitoring. 

**Figure 7:**
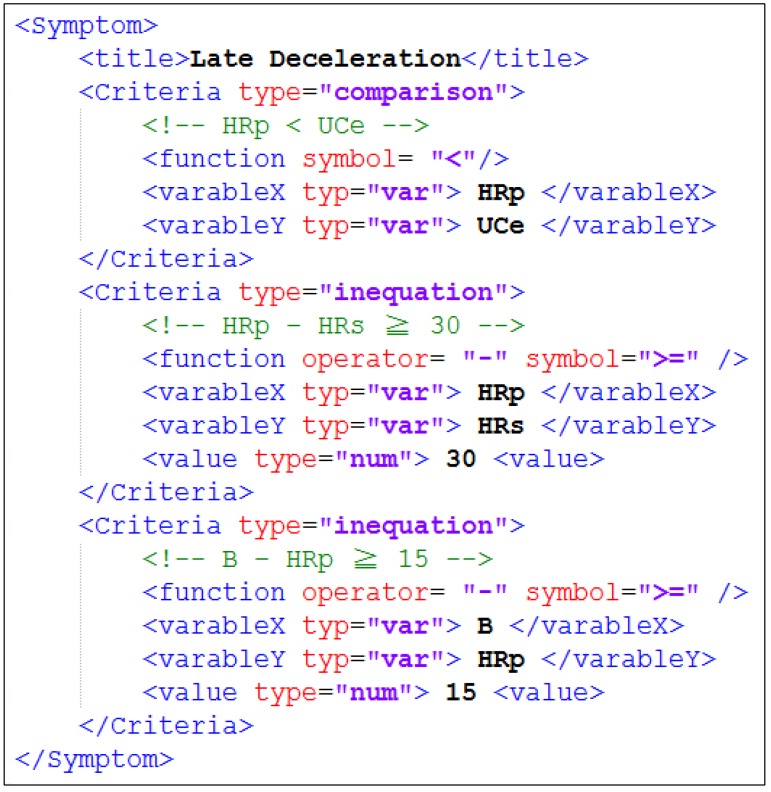
XML document encoding the criteria of “Late Deceleration” symptom.

Diagnostic criteria vary from person to person, depending on numerous factors related to the patient’s physical condition and time to parturition. In IMAIS, the criteria used by the Diagnostic Agent for the diagnosis of a specific gravida are prescribed and maintained by the responsible obstetrician and encoded in XML format. For example, Figure 7 shows the XML file of the criteria for the symptom of “Late Deceleration”. The XML file, along with other personal information, is encapsulated in the “user profiles” repository of the Knowledge-based Data Server. 

#### 3.3.6. External Service

The external services contain the environment hardware and services, including E-mail and SMS. The external service is an extensible module which provides different applications according to different scenarios. If an abnormality is detected in the incoming physiologic fetal data, the Diagnostic Agent initiates external services to inform the associated obstetricians in real-time.

## 4. Diagnostic Engine

Klavan *et al*. [1] noted that “before delivery it is impossible to be fully aware of a fetus at risk”. Examination of medical histories, coupled with observation of changes in fetal heart rate or the mother’s uterine contraction rate present five types of risk symptoms: tachycardia, bradycardia, early deceleration, late deceleration and variable deceleration. The characteristics of these symptoms are described as follows:

(1) *Tachycardia*: Tachycardia refers to a fetal heart rate above average (over 160 bpm). Tachycardia can occur for a variety of reasons, but is generally not directly related to uterine contraction.
(2) *Bradycardia*: Bradycardia refers to a fetal heart rate which is too slow (below 110 bpm). This can have a significant impact on fetal health and can result in death, and thus requires close monitoring.
(3) *Early Deceleration*: Early deceleration yields a curve consistent with uterine contractions, with almost no time lag. The graph drawn by the fetal heart monitor for this symptom shows coinciding periods for the fetal heart rate and the mother’s uterine contractions.
(4) *Late Deceleration*: Late deceleration draws a regular curve mirroring uterine contractions, which is the opposite of early deceleration, presenting an inverted bell curve. A lag consistently occurs at the beginning, peak and end of each contraction.
(5) *Variable Deceleration*: As the name suggests, variable deceleration may assume various shapes (U or V), and is unrelated to uterine contractions. This condition is observed in 80% of birth procedures. Some cases irregular deceleration are related to fetal movements rather than uterine contractions.

**Figure 8:**
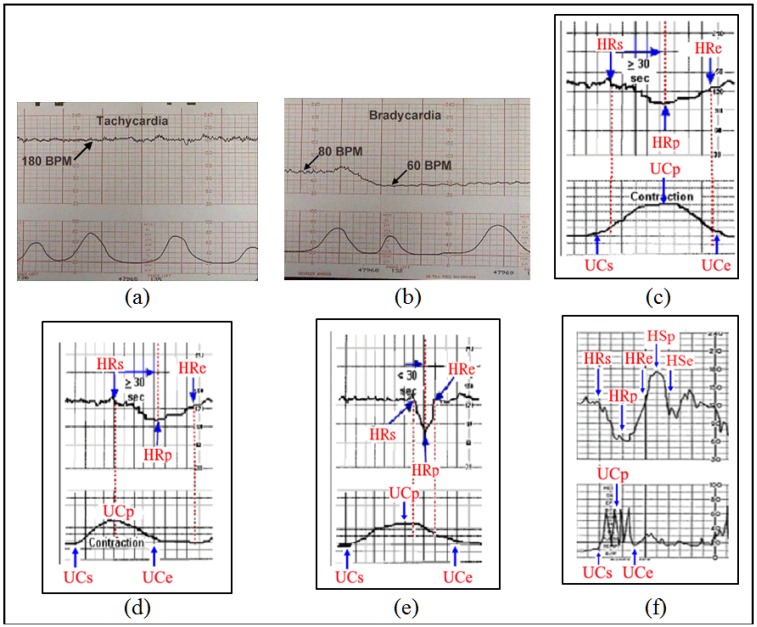
Example patterns of each symptom: (**a**) Tachycardia; (**b**) Bradycardia; (**c**) Early Deceleration; (**d**) Late Deceleration; (**e**) Variable Deceleration—Type A; (**f**) Variable Deceleration—Type B.

The characteristics of these five symptoms are mostly qualitative in nature, thus diagnosis accuracy is highly reliant on obstetrician experience and judgment. Reducing the potential for human error and extending the application of automatic diagnosis requires the development of quantitative guidelines. Prior to the collection of data, we discussed diagnosis procedures of various case histories with the attending obstetrician and establish guidelines for each of the five symptoms. Given that doctors may differ in their diagnostic appraisals, the guidelines are not intended to completely match the standards of each doctor. However, the guidelines are largely uniform, and thus only minor adjustments to rule parameters are required to meet the diagnostic requirements of various doctors in clinical contexts. The diagnosis guidelines are discussed below, with each symptom illustrated with example. The patterns of each symptom are shown in Figure 8 while the rules for detecting abnormalities are illustrated in Table 2. Notations are defined as follows:

- HRs: Heart rate change cycle start time.
- HRe: Heart rate change cycle end time.
- HRp: Nadir in a heart rate change cycle.
- UCs: Uterine contraction cycle start time.
- UCe: Uterine contraction cycle end time.
- UCp: Uterine contraction cycle peak.
- HSp: Peak of the heart rate acceleration cycle (called a “shoulder region” and appearing only in Variable Deceleration).
- HSe: End time heart rate acceleration cycle (called a “shoulder region” and appearing only in Variable Deceleration).
- B: Heart rate baseline.

**Table 2:** The abnormity criteria used in the IMAIS.

Symptom	Criteria
Tachycardia	Heart beat ratio ≥ 160 bpm.
	Condition must last at least three minutes.
Bradycardia	Heart beat ratio ≤ 110 bpm.
	Condition must last at least three minutes.
Early Deceleration	HRp < UCe, *i.e.*, the heart rate reaches its nadir before the uterine contraction cycle is complete.
	HRp – HRs ≥ 30, *i.e.*, over 30 s of lag between the point at which the heart rate begins to drop and its nadir.
	HRs ≥ UCs + 5, *i.e.*, over five seconds of lag between the point at which the heart rate begins to drop and the start of the uterine contraction.
	B – HRp ≥ 15, *i.e.*, the nadir of a heart rate change cycle must be at least 15 beats below the baseline.
Late Deceleration	HRp ≥ UCe, *i.e.*, the heart rate change cycle does not reach its nadir until after the completion of a uterine contraction.
	HRp – HRs ≥ 30, *i.e.*, over 30 s of lag between the point the heart rate begins to drop and its nadir.
	B – HRp ≥ 15, *i.e.*, the nadir of the heart rate change cycle must be at least 15 beats below the baseline.
Variable Deceleration —Type A	HRp – HRs ≤ 30, *i.e.*, less than 30 s of lag between the point the heart rate begins to drop and its nadir.
	HRs ≥ UCs + 5, *i.e.*, the point at which the heart rate begins to drop must lag the beginning of the uterine contraction by at least 5 s.
	B – HRp ≥ 15, *i.e.*, the nadir of the heart rate change cycle must be at least 15 beats below the baseline.
Variable Deceleration —Type B	HRs ≥ UCs + 5, *i.e.*, the point at which the heart rate begins to drop must lag the beginning of the uterine contraction by at least 5 s.
	B – HRp ≥ 15, *i.e.*, the nadir of the heart rate change cycle must be at least 15 beats below the baseline.
	HSp – B ≥ 10, *i.e.*, the peak of the shoulders of acceleration after deceleration must be at least 10 beats above the baseline.
	HSe – HRe ≥ 10, *i.e.*, the lag of the shoulders of acceleration must last at least 10 s.

Fetal heart beats are characterized by irregular ups and downs, thus obstetricians tend to detect actual problems in fetal heart rates only when they are affected by uterine contractions. Thus diagnosing pathologies requires certain data points. 

We obtain the uterine contraction peak (UCp) from a point higher than its two adjacent points, and from the peak we move down to find the intersection with the baseline, which we set as the contraction’s starting (UCs) and ending point (UCe). We then relate the beginning point of the uterine contraction with the fetal heart beat data before searching for the nearest start (HRs) of the heart beat rate drop, the nadir (HRP) and the end (HRe) of the drop. Pathological definitions previously agreed upon with the obstetrician are then applied to complete the diagnosis. 

**Figure 9:**
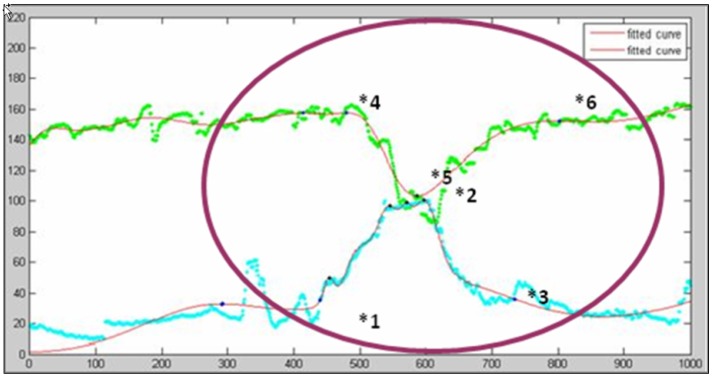
Illustrative example of key points extraction.

Figure 9 provides an illustrative example to describe the key point extraction procedure. The green curve represents the heart rate while the blue curve denotes the uterine contraction. Two fitted curves are also plotted in red.

Since the peak and nadir of the uterine contraction are more regular than the heartbeat, we start the procedure to extract key points from the beginning of the contraction cycle (point *1 in Figure 9) and move forward from there, looking for the peak heart rate (point *4). From here the heart rate begins to drop. Returning to the uterine contraction curve, we move forward searching for the contraction peak (point *2), and then to the end of the contraction cycle (point *3). Starting from the point at which the heart rate begins to fall (point *4), we move forward looking for the nadir (point *5), and the point at which the heart rate stops dropping (point *6), *i.e.*, returning to the baseline level. In this way we collected all the key points required by the diagnostic procedure. We can then apply the rules developed to identify symptoms indicating abnormalities. Each rule is discussed as follows.

- Tachycardia. The heart rate (Figure 9.) is always lower than 160 bpm, thus there is no indication of Tachycardia which would require the heart beat ratio ≥ 160 bpm.
- Bradycardia. The heart rate (Figure 9.) falls below 110 bpm, but only for about 20 s. Bradycardia is only indicated if the heart rate stays below 110 bpm for at least three minutes, and thus can be ruled out.
- Early deceleration. In Figure 9, the fetal heart rate reaches its nadir before the contraction is completed, which meets the criteria for rule 1: HRp < UCe. Since the equipment is set to plot a point twice every second, the distance between the nadir heart rate and the beginning of the fall of heart rate is approximately 90 points, so we assume that the time between the point at which the heart rate began to fall and its nadir is 45 s, which satisfies rule 2: HRp – HRs ≥ 30. The interval between the beginning of the drop in fetal heart rate and the beginning of uterine contraction is over 10 points, meaning that the uterine contraction had already begun at least five seconds before the heart rate began to fall (rule 3: HRs ≥ UCs + 5). Finally, the greatest difference between the baseline and the nadir of the fetal heart rate is approximately 45 beats, which fulfills the requirement of rule 4: B – HRp ≥ 15. Based on the results above, we can determine this is a case of early deceleration.
- Late deceleration. From Figure 9, we can see that the nadir of fetal heart rate occurred before the completion of uterine contraction (violating rule 1: HRp ≥ UCe), so late deceleration can be ruled out.
- Variable deceleration type A. In Figure 9, more than 30 s passed between the beginning of rate decrease to its nadir, thus violating rule 1: HRp – HRs ≤ 30, so Type A variable deceleration can be ruled out.
- Variable deceleration type B. In Figure 9, the interval between the beginning of the drop in fetal heart rate and the beginning of uterine contraction is over 10 points, meaning that the uterine contraction had started more than five seconds before the heart rate began to fall. This satisfies the rule 1 criteria: HRs ≥ UCs + 5. Secondly, the greatest difference between the baseline and the point of nadir fetal heart rate is approximately 45 beats which also fulfills the requirement of rule 2: B – HRp ≥ 15. However, no shoulder region can be found in the heart beat change cycle, thus we can rule out Type B variable deceleration.

This diagnosis procedure successfully identifies symptoms of early deceleration. Doctors are then notified to respond appropriately.

## 5. Usage Scenarios

The proposed platform distinguishes two types of users: (1) medical staff (obstetricians or midwives) and (2) pregnant women whose babies need monitoring. In this section, we describe usage scenarios from these two types of users.

### 5.1. Usage Scenario 1: Medical Staff Perspective

**Figure 10:**
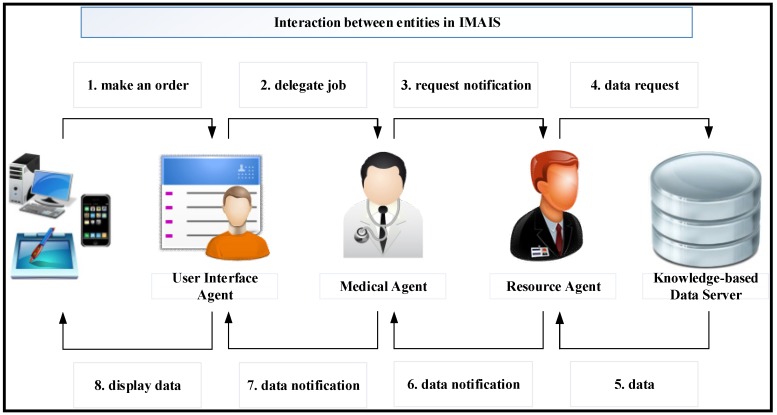
Usage scenario from the perspective of medical staff.

The obstetrician wants to perform a routine check on his patients (gravidas). Through a user interface on his PDA or mobile phone, he requests the UIA (step 1 of Figure 10) collect his patient’s recent health data. Upon receiving the request, the UIA launches and delegates the job to the Medical Agent via the MA network (step 2 of Figure 10). The obstetrician may then switch off his device and proceed with his other work. In the meantime, the Medical Agent surfs the logical MA networks to acquire the needed information. When a container with the needed information is found, the Medical Agent asks the Resource Agent to retrieve the data (step 3 of Figure 10).

The Medical Agent continues delivering messages until it has searched all relevant network nodes. After receiving the request notification, the Resource Agent sends an SQL request for the relevant data(step 4 of Figure 10). The Knowledge-based Data Server sends the data set back to the Resource Agent (step 5 of Figure 10). The Resource Agent sends the query results back to the Medical Agent (step 6 of Figure 10). When the Medical Agent has collected all the required data, it returns to the client-container which created it, and sends the data to the UIA (step 7 of Figure 10). Finally, the UIA displays the data on the application GUI (step 8 of Figure 10) when the obstetrician comes back online.

**Figure 11:**
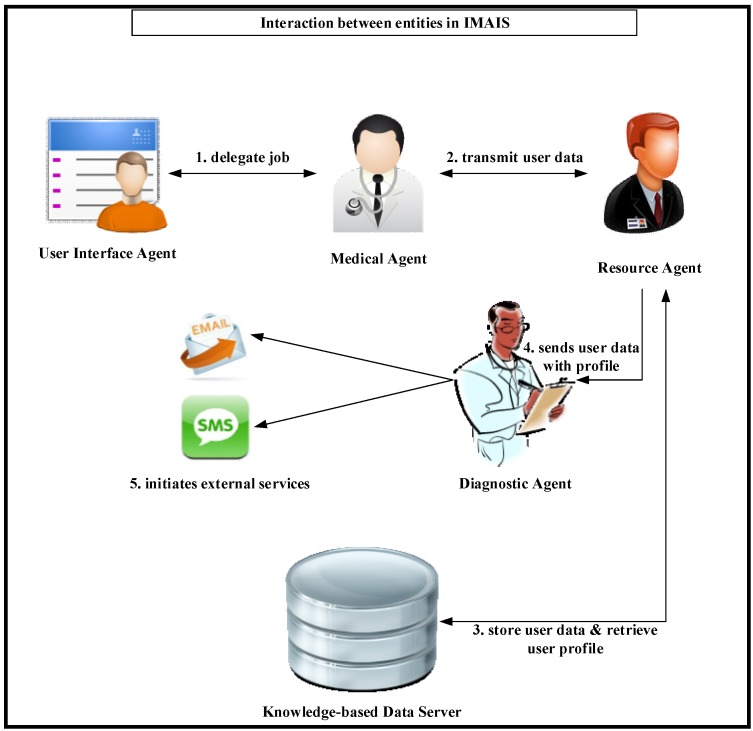
Usage scenario from the user’s perspective.

### 5.2. Usage Scenario 2: User Perspective

A gravida is watching television at home. She turns on her portable fetal monitor to check the status of her baby (FHR and UC). She then initiates her UIA via PDA or smartphone and registers the UIA in the client-container. The UIA then launches and delegates the job to a Medical Agent via the agent network (step 1 of Figure 11). The Medical Agent transmits the fetal data to the Resource Agent of a host in the agent network (Step 2 of Figure 11). The Resource Agent stores the data in the Data Server while simultaneously retrieving the gravida’s personal profile (step 3 of Figure 11). It then sends a copy of the data along with the profile to the Diagnostic Agent (step 4 of Figure 11). The data is then checked against the profile’s criteria for fetal abnormality. If an abnormality is detected, the Diagnostic Agent uses external serves (*i.e.*, SMS, cellular phone or E-mail) to immediately notify the responsible obstetrician(s) and suggests suitable follow-up procedures based on a history of medical treatment (step 5 of Figure 11).

## 6. Implementation

**Figure 12:**
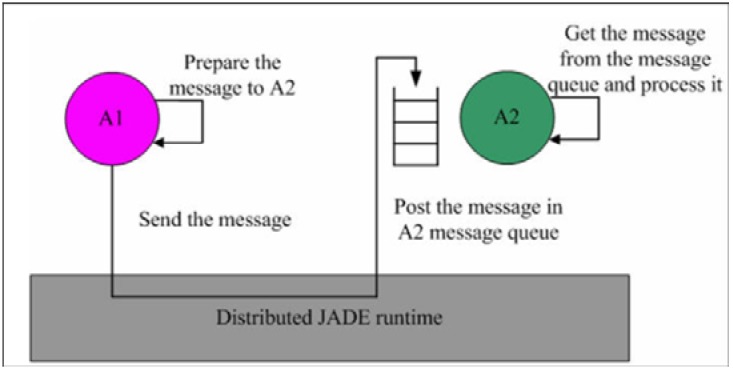
JADE asynchronous message passing paradigm [29].

A fundamental characteristic of multi-agent systems is that individual agents communicate and interact with each other through the exchange of messages. Thus, agents must agree on the format and semantics of these messages. The messages exchanged in the IMAIS agent environment use a format specified by Agent Communication Language (ACL) defined by the FIPA international standard for agent interoperability [25]. Each agent uses a message queue as its mailbox and all messages sent to it from other agents are posted in the mailbox (see Figure 12). The agent is immediately notified of all incoming messages. However, the agent’s developer determines when the agent accesses the messages and how they are processed [29]. 

**Figure 13:**
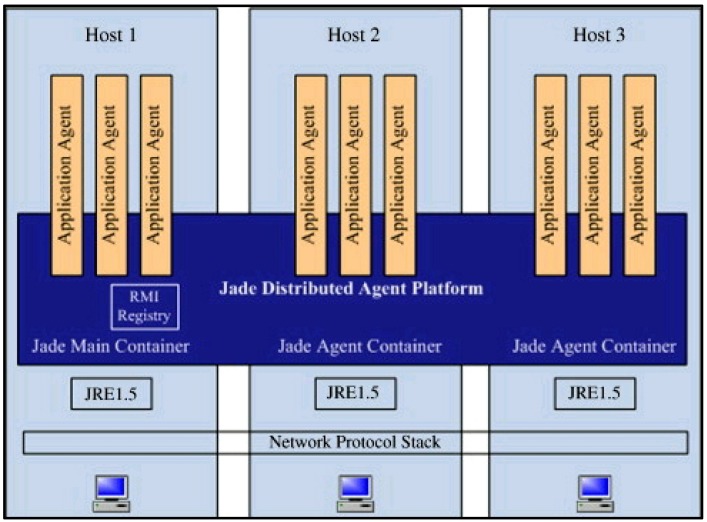
Distributed architecture of JADE [29]

JADE comprises one or multiple Agent Containers residing in the separate Java Virtual Machine and communicating via the Remote Method Invocation (RMI) registry. The main-container, or front-end, hosts the Agent Management System (AMS) agent and Directory Facilitator (DF) agent and creates the platform’s RMI registry. The architecture of the distributed JADE is illustrated in Figure 13 [29].

This section describes the development of the IMAIS prototype in which the system design concept was implemented and tested using JADE. The system prototype was developed in the ERP/MC laboratory at Yuan Ze University in Taiwan. JADE is open, FIPA-compliant and highly-distributed, and can be downloaded from http://jade.tilab.com/. MySQL Community Server versions 5.1 along with Java Database Connectivity (ODBC) for MySQL were installed to develop the knowledge-based data server and to connect the application and database. The JADE-based IMAIS was built on the Windows Server 2003 platform and implemented in Java. JADE requires the Java 2 Run-time Environment (JRE) to run, and the development and compiling of IMAIS agents in JADE required the installation of the full Java 2 Source Development Kit (J2SDK). 

**Figure 14:**
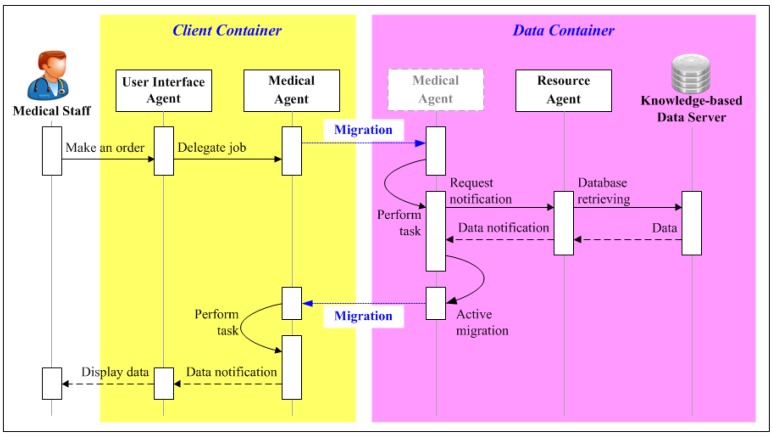
Agent Interactions in IMAIS implementation from the perspective of medical staff.

**Figure 15:**
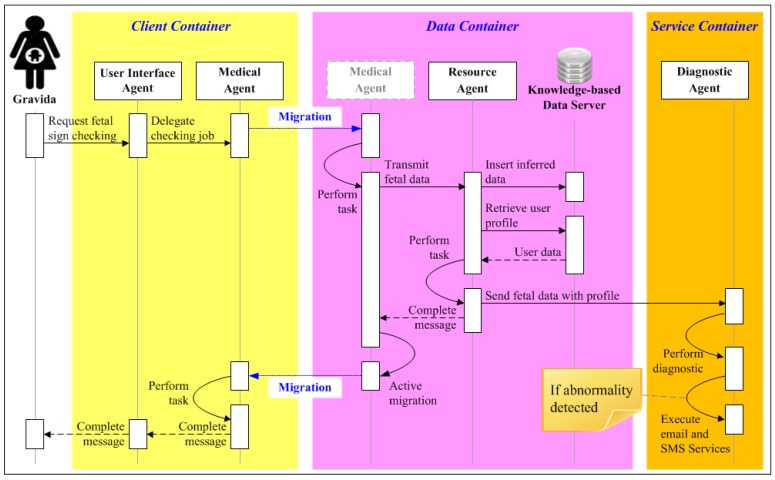
Agent interactions in IMAIS implementation from the perspective of the gravidas.

On the server side, JADE data-containers and Knowledge-based Data Server are used to store user profiles and fetal monitoring records. The Medical Agent moves between the client side and the server side to retrieve fetal monitoring data from all data-containers according to specified date range. After obtaining all required data, the agent returns to the container from which it was originally dispatched and informs the obstetrician that the data is ready. As soon as the user completes fetal monitoring, the data is stored in Knowledge-based Data Server and the Resource Agent passes it to the Diagnostic Agent along with the patient’s personal profile. The interactions between agents and the Knowledge-based data server for medical staff and gravidas are respectively illustrated in Figure 14 and Figure 15.

## 7. Results and System Evaluation

IMAIS for EFM offers obstetricians and midwives an important diagnostic tool in time-critical situations, enabling them to make vital decisions faster and reduce the incidence of expensive false negative alerts. Fetal safety will be improved by immediate communication and exchange of real time data. Confidential patient data are maintained in secure data repositories which are accessible only by authorized personnel. To ensure safe and ethical practices, future modifications will include a more robust security mechanism to meet legal confidentiality requirements. 

The use of MAs allows for direct correspondence between the medical staff and gravidas. In IMAIS, the Medical Agent and Diagnostic Agent serve as medical assistants that relieve hospital personnel from continuous data monitoring tasks and to provide women with a more sensitive and cost-effective solution for close monitoring, without the need for repeated hospital visits. 

The fetal heart rates were recorded in intrapartum deliveries using the ultrasonic Doppler fetal heart signal with fetal monitors manufactured by different companies. The cardiotocogram was also recorded externally. The prototype, IMAIS, was applied to 20 intrapartum labor cases. Two output formats were used: traditional paper printout and digital data. Microsoft Excel^®^ was used to generate a curve from the set of digital data points which was then compared with the original image of the paper graph to validate data correctness and precision.

Different hospitals employ a wide range of equipment, thus data for this research was collected in both digital and traditional paper graph form. Digital data could be directly entered into the diagnostic program to perform symptom diagnosis. Graph printouts, on the other hand, had to be preprocessed into a digital format before the data could be entered into the program for processing. The digitalized graph printouts were used to correlate with our digital format. 

Twenty intrapartum cases with known outcomes were recorded by our IMAIS for Ubiquitous Fetal Monitoring. Sixty-five abnormal fetal heart rates were detected and, in each case, the obstetrician was informed correctly via his or her mobile phone. One late deceleration detected by traditional paper printout diagnostics was missed by our IMAIS diagnostics. 

We applied the National Institute of Child Health and Human Development (NICHD) Guidelines on Fetal Heart Rate Monitoring to a sample of 20 women. Table 3 shows the results obtained from the clinical trials. The resulting data indicated that IMAIS sounded alarms for all abnormalities, but 2% of alerts were false-positives. Diagnosed pattern error was due to poor data quality (signal loss or noise).

**Table 3:** Summary of fetal heart rate monitoring.

	Deceleration	Tachycardia	Bradycardia	Total
Doctor diagnostics	47	16	2	66
IMAIS diagnostics	46	16	2	65
Mobile alarm	46	16	2	65

System usability/readability was evaluated, respectively from the perspective of care-providers and care-recipients through questionnaires conducted with a group of medical staff and a group of gravidas. The medical staff group consisted of gynecology and obstetrics department staff with certain levels of medical experience and computer literacy. The gravida group consisted of twenty women in various stages of pregnancy. The user profiles of the subject groups are illustrated in Table 4 and Table 5.

**Table 4:** User Profiles (Medical Staff).

Personal Information	Question Code	%
*Age*		
30–40	13	65.0
40–50	7	35.0
Total	20	100.0
*Gender*		
Male	12	60.0
Female	8	40.0
Total	20	100.0
*Service year*		
2 or below	5	25.0
2–5	13	65.0
5 or above	2	10.0
Total	20	100.0
*Experience of using Internet*		
Yes	20	100.0
No	0	0.0
Total	20	100.0
*Fluency in using Mobile Devices*		
Yes	19	95.0
No	1	5.0
Total	20	100.0

**Table 5:** User Profiles (Gravida).

Personal Information	Question Code	%
*Age*		
25–35	11	55.0
35–45	8	40.0
45 or above	1	5.0
Total	20	100.0
*The first pregnancy*		
Yes	8	40.0
No	12	60.0
Total	20	100.0
*Experience of using Internet*		
Yes	16	80.0
No	4	20.0
Total	20	100.0
*Fluency in using Mobile Device*		
Yes	14	70.0
No	6	30.0
Total	20	100.0

**Figure 16:**
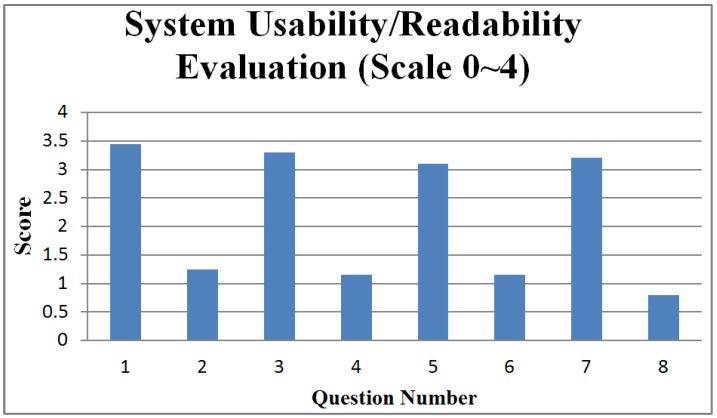
System usability/readability evaluation (medical staff group).

**Figure 17:**
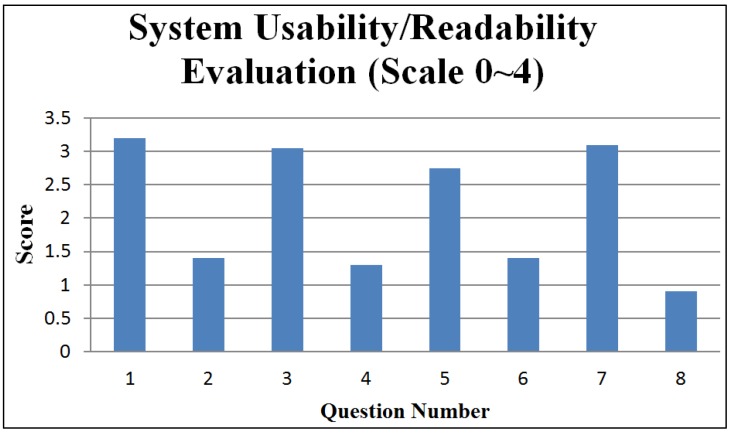
System usability/readability evaluation (gravida group).

In our questionnaire, each question is usually followed by a reversed question to reveal facts from two opposite perspectives. Figure 16 and Figure 17 respectively present statistical summaries of usability/readability for the medical staff group and gravida group. The figures indicate that the usability/readability of the IMAIS is reasonably high.

The question codes in Figure 16 (medical staff group) are keyed to the following questions:

1. IMAIS was easy to use.
2. I would need a technical person to assist me.
3. IMAIS was helpful in promoting home-based fetal monitoring service.
4. IMAIS was not very helpful in promoting home-based fetal monitoring service.
5. IMAIS has helped me in reducing the cases of misdiagnosis.
6. IMAIS has not made much difference in reducing the cases of misdiagnosis.
7. IMAIS has helped me save time spent in fetal monitoring.
8. IMAIS has not helped me save time spent in fetal monitoring.

The question codes in Figure 17 (gravidas) are keyed to the following questions:

1. IMAIS was easy to use.
2. I would need a technical person to assist me.
3. IMAIS provides sufficient functions for my continuous long-term fetal monitoring needs.
4. IMAIS still has a lot room for improvement in terms of its functionality.
5. IMAIS helped me significantly in performing home-based fetal monitoring and in reducing the number of hospital trips.
6. IMAIS has not made much difference in performing home-based fetal monitoring and in reducing the number of hospital trips.
7. IMAIS gave me a better understanding of my baby’s development.
8. IMAIS was not very helpful in helping me better understand my baby’s development.

## 8. Conclusions

Mobile multi-agent systems have great potential to transform the process, and possibly even the outcome, of EFM. The next reasonable step is to evaluate the pilot trial in designing an open and scalable monitoring platform that allows for the direct incorporation of advanced modules in EFM and to smoothly expand EFM coverage areas. For example, the Diagnostic Agent plays a key role in IMAIS but the simple guidelines are still insufficient to perform the accurate and reliable diagnoses. Incorporating sophisticated diagnostic methodologies into IMAIS can effectively enhance the functionality of the Diagnostic Agent. As an essential extension of this work, we will explore the robust diagnostic algorithm in an attempt to achieve the desired level of diagnostic accuracy. 

Another critical issue that remains to be addressed is system security. While mobility facilitates the development of distributed health care systems and allows access for more potential users, it also leaves the system more vulnerable to malicious attacks. JADE is equipped with a basic built-in security mechanism, but the MA is a trust-based paradigm: application security has far-reaching implications and security issues will hinder application acceptance. Robust agent security frameworks [30,31,32,33] for controlling risk and mitigating the threat posed by malicious entities need to be explored and integrated in IMAIS.
